# More Patients or Healthier Populations? Institutionalised Conflict of Interest, Health System Governance, and Population Health

**DOI:** 10.34172/ijhpm.10079

**Published:** 2026-08-24

**Authors:** Aidin Aryankhesal, Hossein Bouzarjomehri, Esmaeil Hosseinzadeh Roknabadi, Roghayeh Mohammadibakhsh, Neil Arya

**Affiliations:** ^1^School of Health Sciences, Faculty of Medicine and Health Sciences, University of East Anglia, Norwich, UK.; ^2^National Centre for Health Insurance Research, Tehran, Iran.; ^3^School of Health Management and Information Sciences, Iran University of Medical Sciences, Tehran, Iran.; ^4^Department of Management Development, Resources and Planning, Hamadan University of Medical Sciences, Hamadan, Iran.; ^5^School of Public Health Sciences, University of Waterloo, Waterloo, ON, Canada.; ^6^Department of Family Medicine, McMaster University, Hamilton, ON, Canada.

**Keywords:** Conflict of Interest, Health Policy, Governance, Health Systems, Corruption, Iran

## Abstract

Conflict of interest (CoI) in health systems is commonly viewed as an individual ethical issue but may also become institutionalised when governance arrangements align policy-making authority with professional or financial self-interest. Using Iran as an illustrative case, this article examines how institutionalised CoI can gradually shape policy priorities and distort health system missions towards provider-centred rather than population-centred goals, often without deliberate intent. A comparative analysis of health ministers’ professional backgrounds suggests substantial variation across countries in governance arrangements and leadership models. The article discusses how institutionalised CoI may contribute to policy distortions including persistent reliance on fee-for-service (FFS) payment, weakened gatekeeping, dual practice, and inadequate transparency, ultimately affecting healthcare utilisation, financial protection, and health system performance. Addressing these challenges requires governance reforms that strengthen leadership, align provider incentives with population health, and improve transparency, accountability, and institutional independence.

## Introduction

 This article examines how conflicts of interest (CoIs) at senior levels of health system governance can gradually become institutionalised, leading policy-making to favour particular professional or financial interests over population health goals, often without deliberate intent. Such distortions can influence financing, service delivery, and regulation, ultimately affecting the health and well-being of entire populations.

 Iran is used as an illustrative case because its integrated health system and policy-making structures have historically been characterised by the sustained influence of clinical professional groups. The authors’ familiarity with the Iranian health system through previous research also enables a deeper examination of these governance arrangements. However, similar institutional dynamics can be observed, to varying degrees, across many health systems. Clinical expertise remains indispensable to policy-making; the concern is not clinicians in leadership but governance arrangements that concentrate authority within groups whose secondary interests are directly affected by the policies they formulate.

## Conflict of Interest, Individual, and Institutional

 CoI refers to situations in which a secondary interest, such as personal, financial, professional, political, or ideological gain, risks unduly influencing primary obligations, including the protection of public welfare.^1^ An *interest *is any commitment, incentive, or value that may shape an individual’s judgement or behaviour. A conflict arises when such secondary interests have the potential to compromise impartial decision-making or the fulfilment of primary responsibilities.

 CoI may be individual, where private interests bias professional judgement, or institutional, where organisational or structural incentives conflict with public missions. Institutionalised CoI arises when governance arrangements embed and normalise such conflicting incentives. In this context, it refers not merely to organisational profit-seeking, but to the structural entrenchment of professional dominance within decision-making systems, whereby policy-makers’ identities and career trajectories align self-serving incentives with public authority. This interpretation is consistent with broader analyses of decision-making, which highlight how self-interest and cognitive biases can systematically distort ostensibly objective policy choices.^2^ This concept is related to Lessig’s notion of institutional corruption, in which influences within an institution’s “economy of influence” can undermine its effectiveness and public trust.^3^ However, we use institutionalised CoI here to focus specifically on how competing professional and financial interests become embedded within health-system governance.

 Institutionalised CoI is not unique to clinicians. Politicians, public administrators, insurers, industry representatives, and other stakeholders may also hold financial, professional, political, or ideological interests capable of influencing policy decisions. The concern is therefore not the professional background of policy-makers itself, but governance arrangements that concentrate decision-making authority among groups whose secondary interests are directly affected by the policies they formulate and implement. In the Iranian context, the longstanding dominance of specialist physicians provides an illustrative example of how such institutionalised conflicts may emerge.

 Institutionalised CoI is particularly damaging when it affects health system stewardship and governance. When CoIs shape this core function, policy perspectives become short-sighted, reform agendas are distorted, and health systems may become increasingly captured by powerful interest groups. In some countries, including Iran, this dynamic is reflected in the recurrent appointment of ministers and senior officials drawn from clinical specialties, some of whom maintain direct or indirect financial ties to private healthcare enterprises.^4^ This pattern reflects not isolated ethical lapses, but governance arrangements that may systematically privilege professional self-interest over population health goals. While Iran is used here as an illustrative case, similar governance dynamics can be observed to varying degrees across a range of health systems.

## Iran as an Illustrative Case

 Iran provides an informative case because its Ministry of Health and Medical Education combines national policy-making, regulation, medical education, and delivery of much of the public health system through affiliated universities and hospitals.^5^

 Historically, Iran’s health system has evolved within this integrated governance structure into a physician-led hierarchy, in which prestige, professional status, and proximity to political power have often influenced advancement to senior leadership positions. Over time, these arrangements may contribute to the institutionalisation of CoIs within the government structure. In contrast, management theory emphasises that senior leadership roles require conceptual, political, and administrative capabilities, in addition to technical expertise.^6^ Consequently, persistent concentration of leadership within a single professional group may constrain meritocratic advancement, weaken governance capacity, and reinforce a treatment-oriented policy perspective. In this sense, professional dominance functions not merely as a sociological characteristic, but as a mechanism through which institutionalised CoI may be reproduced and legitimised.

 A brief comparative analysis suggests that countries differ in the professional backgrounds of their health ministers, with some more frequently appointing clinicians and others selecting leaders from public policy, social sciences, law, or other professional backgrounds (Figure 1). The purpose of this comparison is not to suggest that physicians are inherently less capable health ministers or that professional background alone determines policy outcomes. The experience of new public management reforms also illustrates why leadership background alone cannot determine whether governance advances population health. Although new public management sought to improve efficiency and accountability, evidence from health systems shows that its implementation could produce unintended effects, reinforcing the importance of institutional design and accountability rather than leadership background alone.^7^ In many countries, even when ministers have clinical training, they are no longer engaged in active clinical practice and instead pursue careers in policy-making or public administration. However, in some contexts, including Iran, close links between clinical practice, professional networks, and health sector governance may persist. Such links can increase the risk that governance structures are shaped by the same professional groups they are intended to regulate, thereby reinforcing institutionalised CoIs. While institutionalised CoI may arise from multiple sources, governance systems with stronger institutional separation between policy-making and professional interests are generally better positioned to identify and manage these risks.

**Figure 1:**
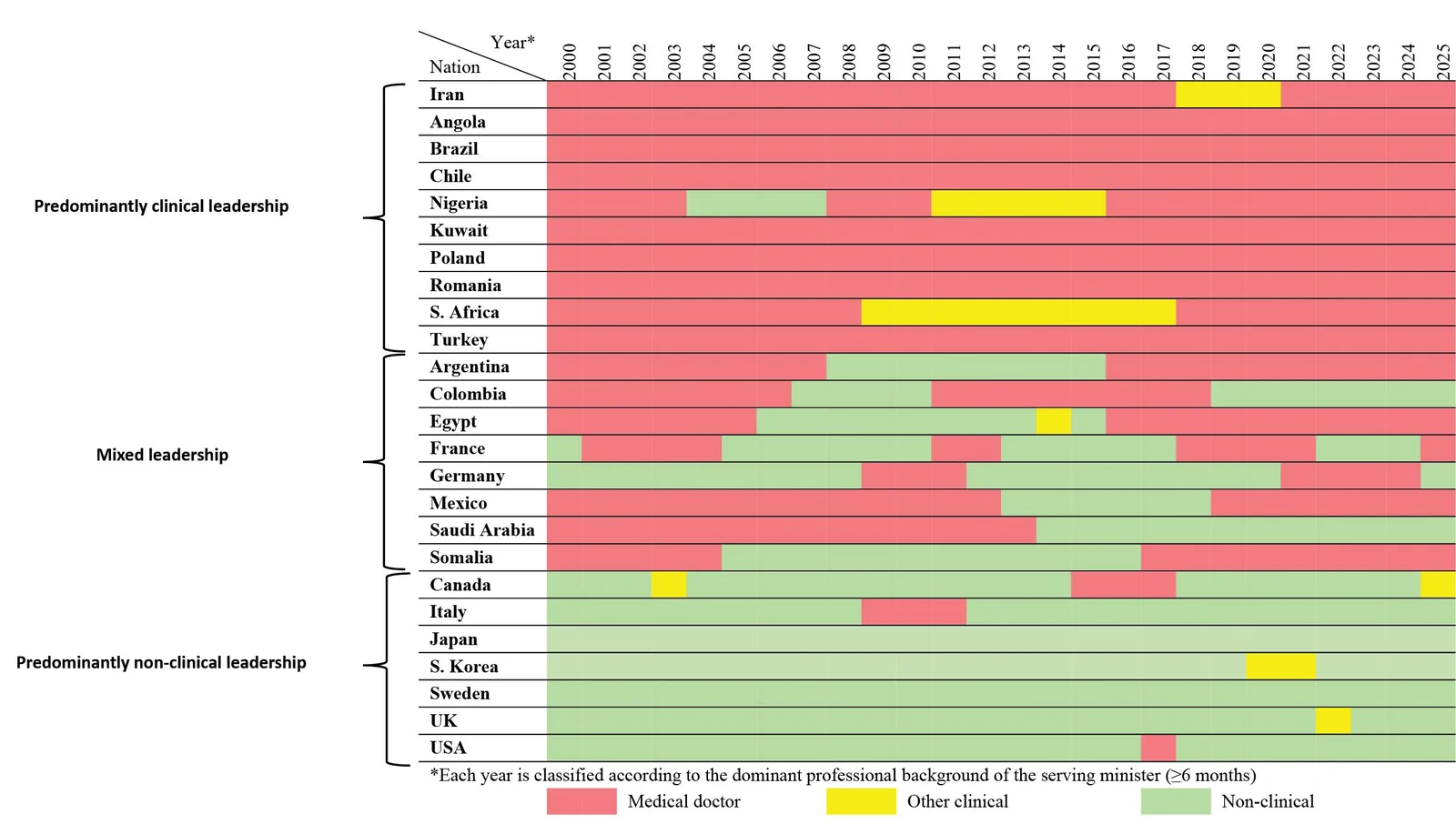
Professional Backgrounds of Health Ministers (2000–2025): Clinical and Non-clinical Leadership Patterns.

## Policy Distortions Driven by Institutionalised Conflict of Interest

 Institutionalised CoIs influence health systems by shaping governance structures, policy priorities, and incentives over time.^8^ Evidence from diverse settings suggests that overlapping professional, financial, and institutional interests can affect agenda setting, policy formulation, implementation, and regulatory oversight, often without deliberate misconduct.^9^ Weak governance arrangements allow these interests to become embedded within health systems, increasing the likelihood that policies favour particular professional or commercial interests over broader population health goals. Iran provides an illustrative example of these broader governance dynamics, where the long-standing influence of specialist groups in senior policy-making positions may contribute to a cascade of policy distortions through aligned professional and financial incentives.^8,10^

###  Provider Payment Incentives

 Fee-for-service (FFS) remains one of the most common provider payment methods, rewarding clinicians according to service volume. Excessive reliance on FFS can generate moral hazard, incentivise unnecessary care, and contribute to policy failures.^11^ In Iranian public hospitals, FFS payments constitute a major source of income for some physicians and may exceed their fixed salaries. Where professional groups substantially influence policy-making, reforms to alternative payment models may encounter institutional resistance because existing arrangements align provider income with service utilisation. Consequently, FFS can reinforce institutionalised CoIs by preserving financial incentives that favour treatment volume over population health objectives.

###  Weakening of Gatekeeping 

 Effective gatekeeping promotes appropriate referrals, continuity of care, and efficient resource allocation. However, under FFS payment systems, unrestricted access to specialist services may increase provider income while weakening the role of primary care.^12^ Consequently, reforms that strengthen referral pathways or expand family physician programmes may face institutional and political resistance where professional interests depend on specialist-led service delivery.

###  Self-governing Public Hospitals

 Greater managerial autonomy can improve hospital efficiency, but where financial sustainability depends largely on FFS revenue, it may also encourage higher service volumes and more complex interventions. If policy-makers remain closely connected to provider organisations, reforms that reduce these incentives may prove difficult, allowing institutional CoIs to persist.

###  Dual Practice 

 Simultaneous employment in public and private sectors is common in many health systems and may support workforce retention. However, where governance safeguards are weak, dual practice can create competing incentives between public responsibilities and private income. In countries such as Iran, higher private-sector tariffs combined with FFS payments may encourage movement of patients between sectors and reinforce institutional CoIs.^13^

###  Transparency and Disclosure

 Transparent governance and effective disclosure are essential safeguards against institutionalised CoIs. Weak disclosure of financial or professional interests among policy-makers, regulators, and healthcare organisations increases the risk of regulatory capture and undermines public trust.^14^ Strong governance therefore requires systematic identification, disclosure, and management of competing interests.

## Consequences for Healthcare Delivery

 These governance arrangements affect healthcare delivery, utilisation, financing, and financial protection. The following examples illustrate these broader consequences using evidence from Iran.

###  Supplier-Induced Demand

 Supplier-induced demand is a well-recognised consequence of misaligned provider incentives. When providers or decision-makers have direct financial interests in service provision, incentives to increase consultations, investigations, procedures, or prescriptions may outweigh incentives to optimise patient outcomes. Empirical evidence from Iran indicates that a considerable proportion of diagnostic imaging, laboratory investigations, and other healthcare services may be delivered without clear clinical indications and may be influenced by financial relationships among providers.^15^ Similar patterns have been reported in many other health systems, demonstrating that supplier-induced demand is not unique to Iran but represents a broader consequence of governance arrangements that fail to adequately manage CoIs.^16^

###  Out-of-Pocket Payments and Catastrophic Health Expenditures

 Institutionalised CoIs may also increase the financial burden placed on patients and households. Payment systems that reward service volume, together with supplier-induced demand and weak regulatory oversight, contribute to higher healthcare utilisation and increased out-of-pocket expenditure.^17^ In Iran, National Health Accounts estimate that direct household payments account for approximately 48% of total health expenditure, with physician income remaining closely linked to service volume.^18^ Evidence from the Iranian Parliament Research Centre further suggests that households receiving care within settings characterised by unmanaged CoIs experience a substantially higher risk of catastrophic health expenditure than other patients.^19^

 High out-of-pocket expenditure reduces equity, discourages timely access to care, and undermines progress towards universal health coverage, illustrating how governance failures translate into poorer health system performance.

Figure 2 summarises the conceptual pathway proposed in this editorial, illustrating how institutionalised CoIs embedded within governance structures can influence policy decisions, shape provider incentives, generate distortions in healthcare delivery, and ultimately affect health system performance and financial protection.

**Figure 2:**
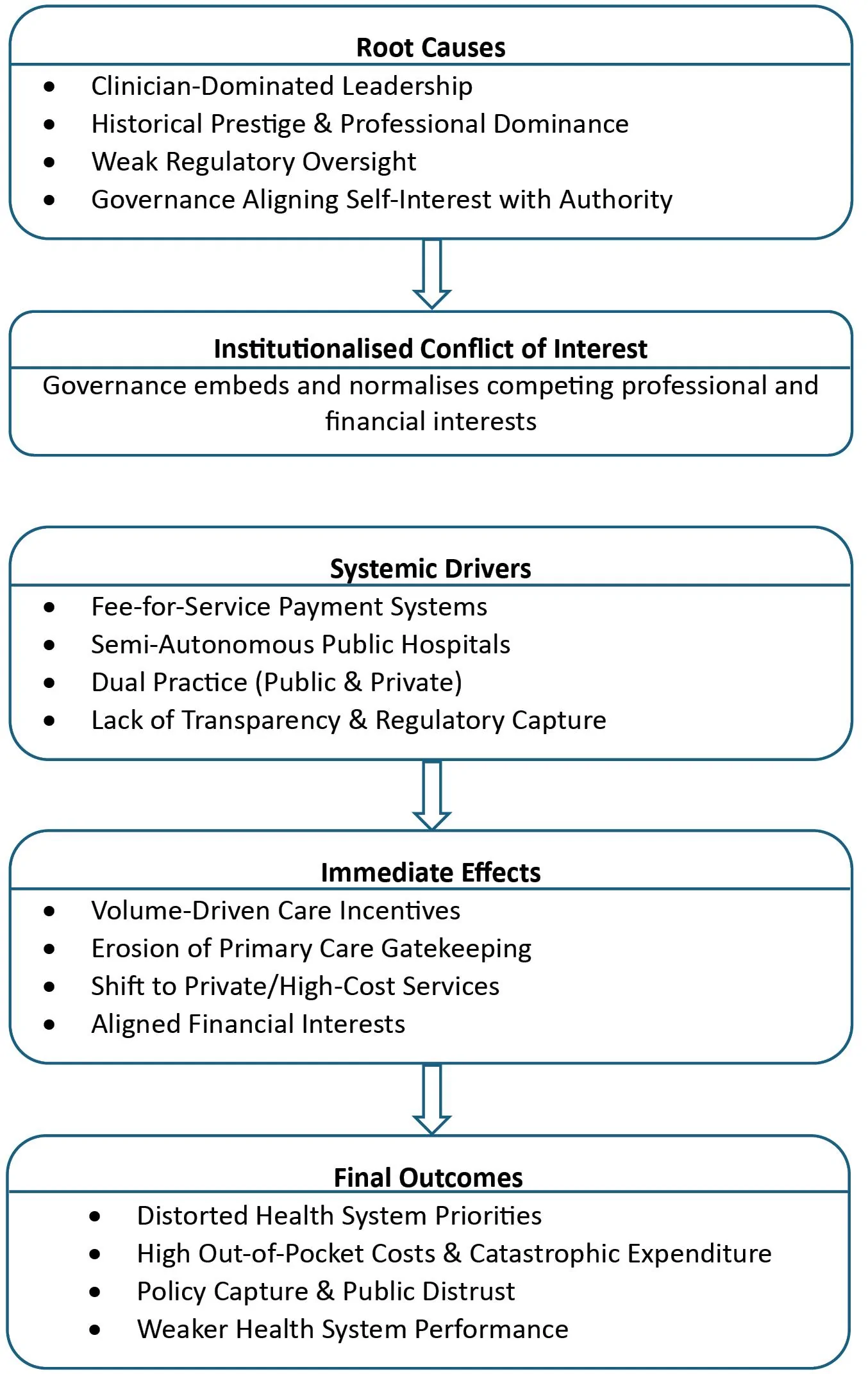
Conceptual Pathways for the Emergence and Effects of Institutional Conflict of Interest in Health Systems.

## Priority Actions to Address Institutionalised Conflict of Interest

 Health systems are vulnerable to CoIs because they involve complex relationships among governments, healthcare professionals, insurers, industry, and patients. Where safeguards are weak, these conflicts may become embedded within governance arrangements, increasing risks of regulatory capture, policy inertia, and declining public trust. Addressing these challenges requires governance reforms that strengthen transparency, accountability, and institutional independence rather than technical reforms alone.^20^

 Four priority actions are therefore essential:

###  Reform Leadership and Governance Structures

 Policy-making authority should be supported by governance arrangements that minimise institutional CoIs while ensuring that technical expertise continues to inform decision-making. Transparent appointments, multidisciplinary leadership, and independent oversight strengthen public trust and stewardship.

###  Redesign Payment and Incentive Systems

 Provider payment systems should reward quality, value, and population health outcomes rather than service volume alone. Where FFS mechanisms remain necessary, blended payment models and safeguards should be considered to reduce incentives for unnecessary care.

###  Clarify Boundaries Between Public and Private Sectors

 Appropriate governance mechanisms are needed to manage competing interests associated with dual practice and interactions between public and private sectors. These may include clearer accountability arrangements, transparent regulation, and institutional safeguards that minimise incentive spillovers while maintaining workforce sustainability.

###  Strengthen Transparency, Disclosure, and Accountability Mechanisms

 Transparent governance and effective accountability are essential safeguards against institutionalised CoIs. Health systems should establish standard mechanisms to identify, disclose, and manage financial and non-financial CoIs among policy-makers and other key stakeholders. Disclosure alone is insufficient; governance frameworks should specify management strategies such as recusal, independent oversight, or reassignment. Institutional transparency should also extend to policy-making, procurement, and relationships with commercial interests to reduce regulatory capture and strengthen public trust.

 Ultimately, institutionalised CoI can shift health system priorities away from prevention and primary care towards curative and specialist care, favouring greater service utilisation over the prevention of illness and healthier populations.

## Disclosure of artificial intelligence (AI) use

 Section(s) of manuscript affected: Entire manuscript.

 Type of AI tool(s) used: ChatGPT.

 Purpose of use: Language editing.

## Ethical issues

 Not applicable.

## Conflicts of interest

 Authors declare that they have no conflicts of interest.
